# *Lavandula stoechas* (L) a Very Potent Antioxidant Attenuates Dementia in Scopolamine Induced Memory Deficit Mice

**DOI:** 10.3389/fphar.2018.01375

**Published:** 2018-11-23

**Authors:** Aamir Mushtaq, Rukhsana Anwar, Mobasher Ahmad

**Affiliations:** ^1^Department of Pharmacology, Punjab University College of Pharmacy, University of the Punjab, Lahore, Pakistan; ^2^Gulab Devi Institute of Pharmacy, Gulab Devi Educational Complex, Lahore, Pakistan

**Keywords:** dementia, neurodegeneration, AChE, *L. stoechas*, elevated plus maze

## Abstract

The objective of the current project was to explore the pharmacotherapeutic role of *Lavandula stoechas* (L) for the management of dementia. Dementia is considered a global challenge of current century seeking special attention of pharmacologists to explore its best remedies. Methanolic extract of aerial parts of *L. stoechas* was tested for phytochemical analysis along with free radical scavenging activity. Behavioral studies were performed on scopolamine induced amnesic mice by using elevated plus maze (EPM), light and dark test and hole board paradigms. Biochemical investigations were made after decapitating the mice. Their brains were isolated for biochemical estimation of acetylcholinesterase (AChE), malondialdehyde (MDA), superoxide dismutase (SOD), catalase (CAT), and glutathione (GSH). Phytochemical study ensured the presence of total phenolic contents (285.91 ± 0.75 mg of GAE/g of extract), total flavonoids (134.06 ± 0.63 mg of RE/g of extract), total tannins (149.60 ± 0.93 mg of TAE/g of extract) and free radical scavenging activity (IC_50_ value = 76.73 μg/ml found by DPPH method). Behavioral studies indicated that animals of GVII showed higher inflexion ratio (0.40 ± 0.03) for EPM, spent most of time (227.17 ± 2.13 s) in dark area of light dark test and had many hole pockings (39.83 ± 1.88) for hole board paradigm. Moreover, biochemical studies revealed that methanolic extract of *L. stoechas* (800 mg/kg/p.o.) significantly (*P* < 0.001) reduced brain AChE and MDA levels while improved SOD, CAT, and GSH levels. Thus the findings suggest that *L. stoechas* stabilizes memory by enhancing cholinergic neurotransmission and by providing defense against oxidative stress in mice brain.

## Introduction

Dementia results from neurodegenerative insult in brain neurons. Neurodegeneration not only leads to the impairment of memory but also alters the social and behavioral compliance. Cholinergic hypothesis (Francis et al., 1999) describes the pathogenesis of dementia, according to which severity of the disease is coupled with neuronal damages in septohippocampal cholinergic system (Giovannini et al., 1997) associated with learning and cognitions (Ballard et al., 2005). Acetyl cholinesterase inhibitors like donepezil, galantamine, rivastigmine (Bejar et al., 1999) and tacrine can be used for the symptomatic management of mild dementia but they have little therapeutic success because they do not prevent neurodegeneration and are associated with serious adverse effects (Hake, 2001). Herbal remedies are considered more safe, gentle and reliable in enhancing memory and cognitive functions (Bhattacharjee et al., 2015). Roughly, 150 traditional plants and herbs have been used so for, either singly or in combined preparations to stop neurodegeneration in brain (Adams et al., 2007). This is of great worth to identify the active constituent for the management of dementia and cognitive disorders which will definitely enhance the practice of customary herbs in the field of neuropharmacology (Kumar et al., 2012).

*Lavandula stoechas* L. (Lamiaceae) has been extensively used by traditional healers for the management of CNS disorders including epilepsy, dementia, and migraine (Hakeem et al., 1991). This is also known as the broom of the brain. Its aerial parts have been extensively studied for phytochemical work (Gilani et al., 2000). The present research work was aimed to explore the pharmacological basis of enhancement of memory to strengthen the folk and traditional use of *L. stoechas* as a memory enhancer.

## Materials and Methods

### Drugs and Chemicals

Acetic acid (71251), 2,2*-*diphenyl*-*1-picrylhydrazyl (DPPH) (D9132), acetyl thiocholine iodide (A22300), carboxy methyl cellulose (CMC) (419273), 5,5′-dithiobis-2-nitrobenzoic acid (DTNB) (D218200), aluminum chloride (563919), ascorbic acid (A0278), chloroform (C2432), ethanol (E7023), Folin-Ciocalteu reagent (FCR) (F9252), gallic acid (G7384), methanol (34860), *n*-butanol, nitro blue tetrazolium (NBT) (N5514), phenyl methanesulfonate (PMS) (495085), superoxide dismutase (SOD) (S9697), hydrogen peroxide (H_2_O_2_) (16911), potassium acetate (P1190), potassium dichromate (P5271), reduced nicotinamide adenine dinucleotide (NADH) (N8129), rutin (R5143), sodium carbonate (S7795), sodium dodecyl sulfate (436143), tannic acid (403040), trichloracetic acid (TCA) (T6399) and thiobarbituric acid (TBA) (T5500) all were procured from Sigma-Aldrich, Ms Traders, Lahore Pakistan. Piracetam and scopolamine was donated by Jiangxi Yuehua Pharmaceutical, China and Merck Pharmaceutical Pvt. Ltd. Pakistan, respectively, upon special request. All chemicals of analytical grades were used in this research.

### Preparation of Plant Extract

Dried aerial parts of *L. stoechas* were purchased from local market of Lahore, Pakistan and were identified by Department of Botany GC-University, Lahore. Plant specimen was preserved in herbarium with voucher number GC.Herb.Bot.3386. For extraction, dried aerial parts were ground into coarse size powder and 1 kg of it was soaked into 5 L methanol (99.5%) in glass jar for 3 days. It was then filtered first by muslin cloth and then through Whatman No. 1 filter paper. Filtrate was concentrated in buchi rotavapor and residue was again macerated in recovered methanol for another 3 days. In this way, after three consecutive soakings concentrated filtrates were mixed up and dried in dry heat oven at 37°C. Finally dark green colored thick extract was obtained which was labeled after sealing in glass jar and put into refrigerator at 4°C temperature. Percentage yield was calculated as;

% Age yield = Weight of extract (g)/Weight of plant material (g) × 100

### Phytochemical Analysis of Plant Extract

Preliminary phytochemical testing of *L. stoechas* methanolic extract was performed to explore the major phytochemical classes actually responsible for anti-oxidant and anti-amnesic activities. Proteins, carbohydrates, alkaloids, glycosides, flavonoids, steroids, terpenoids, saponins, tannins, phenols, quinones, phytosterol, terpenes, and fixed oil were tested qualitatively (Mushtaq et al., 2013).

#### Estimation of Phenolic Contents

Total phenolic contents of plant extract were determined by using FCR as described by Kumaran and Karunakaran (2007). For determination of total phenols sample solution was prepared by mixing 100 μl of *L. stoechas* methanolic extract (100 μg/ml), 500 μl of FCR and 1.5 ml of 20% sodium carbonate. Solution was shaken vigorously in vortex mixer after making the volume of solution 10 ml by adding distilled water and mixture was incubated for 2 h. Similarly, standard solution was prepared by adding 100 μl of different dilutions of gallic acid (50, 100, 150, 200, 250, 300, 350, and 400 μg/ml) separately in reaction mixture instead of *L. stoechas* extract. Then absorbance of both sample and standard solutions were determined at 765 nm against blank. All readings were taken in triplicates and total phenolic contents in plant extract were expressed as gallic acid equivalent (GAE) by drawing gallic acid calibration curve. Following formula was used to find total phenol contents in plant extract; *C* = *Ci* × *V*/*W*

*C* = total phenolic content in mg/g, *Ci* = concentration of gallic acid established from calibration curve in mg/ml, *V* = volume of extract in ml, and *W* = weight of plant extract in gram.

#### Estimation of Total Flavonoids

Flavonoid contents were found by using aluminum chloride method as proposed by Kumaran and Karunakaran (2007). Stock solutions were prepared separately by taking 0.5 ml of plant extract (1 mg/ml) and standard rutin (10–100 μg/ml). To each test tube, 1.5 ml of methanol, 0.10 ml potassium acetate, 0.10 ml aluminum chloride and 2.8 ml distilled water was added with constant shaking. All solutions were filtered and absorbance was taken at 510 nm. A calibration curve was drawn for rutin and total flavonoid contents were found as mg rutin equivalents (RE)/g dry extract.

#### Estimation of Total Tannins

Tannins in plant extract were determined by modified Folin-Ciocalteu’s method by preparing 0.1 ml standard tannic acid solutions of different dilutions (50, 100, 150, 200, 250, 300, 400, and 500 μg/ml). Sample solution was prepared by taking 0.1 ml of *L. stoechas* methanotic extract (200 μg/ml). To each test tube, FCR (0.5 ml) and 35% sodium carbonate (1 ml) were mixed and final volume was made 10 ml by adding distilled water. All the test tubes were shaken and kept at room temperature for half an hour and absorbance was read at 725 nm against a blank. Tannic acid calibration curve was drawn and total tannin contents of extract were expressed as mg tannic acid equivalent/g of extract (Polshettiwar et al., 2007).

### *In vitro* Antioxidant Activity

#### DPPH Free Radical Scavenging Assay

Antioxidant activity of *L. stoechas* methanolic extract was determined by using DPPH free radical scavenging assay developed by Blois (1958). Methanolic solution of DPPH (1.0 mmol/L) was prepared in volumetric flask, covered with aluminum foil and put into dark place after marking as reagent stock solution. Different solutions of plant extract and standard ascorbic acid (1 ml each) having concentrations (20, 40, 60, 80, 100, and 200 μg/ml) were prepared separately in test tubes. Then, 2 ml of reagent stock solution was put into each test tube, mixed on vortex and incubated for 30 min, at 37°C in dark place. Blank solution was prepared in the same way having all the ingredients except test substances. The absorbance of blank and all test solutions was measured at 517 nm by using UV-Vis spectrophotometer. Percentage scavenging activity was determined by applying following formula:

Radical scavenging (%) = Absorbance of Blank - Absorbance of Sample × 100/Absorbance of Blank

### Experimental Animals

Male Swiss albino mice (20–25 g) were used in this study. They were housed in polycarbonate cages in animal house of Punjab University College of Pharmacy, University of the Punjab, Lahore. Special permission regarding animal ethics was obtained from research ethics committee of the institute with diary number AEC/PUCP/1072. The animals were provided with standard living conditions (temperature; 25 ± 2°C, humidity; 50 ± 5% and 12:12 light/dark span) and had free access of standard pellet diet and water *ad libitum.* Animals were acclimatized in lab and were trained for all three paradigms for 1 week before the start of behavioral experiments.

### Study Design

Mice were divided into seven groups (*n* = 6) and were treated accordingly as shown in Table 1.

**Table 1:** Study design.

No.	Treatment from day 1 to day 7	Treatment on day 7th, 45 min after administration of the last dose
G-I (Normal Control)	Normal saline 10 ml/kg/p.o.	–
G-II (Amnesic Control)	5% CMC 10 ml/kg/p.o.	Scopolamine (10 mg/kg/p.o.)
G-III (Standard Control-A)	Piracetam 200 mg/kg/p.o.	–
G-IV (Standard Control-B)	Piracetam 200 mg/kg/p.o.	Scopolamine (10 mg/kg/p.o.)
G-V (Experimental Control-I)	meL.s 200 mg/kg/p.o.	Scopolamine (10 mg/kg/p.o.)
G-VI (Experimental Control-II)	meL.s 400 mg/kg/p.o.	Scopolamine (10 mg/kg/p.o.)
G-VII (Experimental Control-III)	meL.s 800 mg/kg/p.o.	Scopolamine (10 mg/kg/p.o.)

*Scopolamine and piracetam were dissolved in normal saline while *L. stoechas* was suspended in 5% CMC for dose preparation. Then animals were subjected to elevated plus maze (EPM), light and dark test apparatus and hole board test apparatus for behavioral assessment*.

### Behavioral Studies

All behavioral experiments were performed in sound proof room in dim day light and mice were put apart to avoid acoustic and visual disturbances. Observations were recorded by using digital camera connected with computer monitor.

#### Elevated Plus Maze

Elevated plus maze (EPM) is considered the most reliable paradigm for evaluation of memory (Pahaye et al., 2017), which was made up of two poly acrylic sheets joined together in shape of plus sign in such a way that it had two open arms (16 cm × 5 cm), two closed arms (16 cm × 5 cm × 12 cm) and a central platform (5 cm × 5 cm). Apparatus was put on a wooden stand elevated 25 cm from floor (Kulkarni et al., 2011). Mouse was put on open arm by facing it away from central platform and time taken (s) by it to move in any of closed arm with all its four legs was recorded and then returned into its home cage. Each animal was given maximum 90 s to explore the apparatus. If it failed to find closed arms for given time then it was pushed into closed arm with its tale and latency time was marked as 90 s for that animal. Initial transfer latency was observed after 45 min of administration of scopolamine and retention of latency was recorded after 24 h of administration of scopolamine and inflexion ratio (IR) (Kasture et al., 2007) was calculated by following formula; IR = (L_0_ – L_1_)/L_0_ where, IR = inflexion ratio, L_0_ is initial transfer latency (s) and L_1_ is retention transfer latency in seconds.

#### Light and Dark Test Apparatus

This paradigm is used for the evaluation of learning tasks as proposed by Barry et al. (1987), which is based upon the principle that rodents prefer to live in dark compartment. Apparatus was made up of poly acrylic sheets and had two compartments; larger (30 cm × 30 cm × 35 cm) transparent chamber separated from smaller chamber (20 cm × 30 cm × 35 cm) which was colored black to make it dark. Both chambers had small opening (5 cm × 5 cm) in the middle bottom of separating wall for entrance. The floor of both chambers was marked by lines each 1 mm apart. Mouse was put in the light chamber and was observed carefully for 5 min to record the time spent in each chamber. According to study designs, the animals were given doses and observations were recorded by using light and dark test apparatus for 2 days after the completion of last dose.

#### Hole Board Test

For assessment of learning behavior, hole board test paradigm (Durcan and Lister, 1988) was used with minor modification (Hossain and Uma Devi, 2001) which was composed of rectangular shaped, poly acrylic box (35 cm × 45 cm × 45 cm). Black colored sheet contained sixteen holes (2 cm diameter and at equal distance) was supported on corners, 5 cm above the bottom of box. The animal was put in the middle of box and observed for 5 min to record number of hole pokings. Animals were given doses according to the protocols of study design and then observations were recorded for two consecutive days.

### Biochemical Assessment

After performance of behavioral tests the animals were given anesthesia by using chloroform and were decapitated to isolate brains. Each brain was rinsed with ice cold normal saline after weighing it and 20 mg of it was homogenized in 1 ml ice cold phosphate buffer (pH 7.4.) by using tissue homogenizer. To separate out the nuclear debris, the homogenized mixture was then centrifuged for 5 min at 69.40 ×*g* by keeping the temperature 4°C. Supernatant was again centrifuged for 20 min at 1,0845 ×*g* at the same temperature and supernatant thus obtained was used for biochemical tests (Rajesh et al., 2017).

#### Acetylcholinesterase (AChE) Activity

Ellman’s method was used for estimation of AChE level for which 0.4 ml of supernatant was taken in cuvette already contained 2.6 ml of phosphate buffer (0.1 M/L, pH; 8) and 100 μL of 5,5′-dithiobis-2-nitrobenzoic acid. The reaction mixture was thoroughly mixed and absorbance was recorded several times by using UV-Vis spectrophotometer at 412 nm. Then 20 μL of acetyl thiocholine iodide was added as substrate in the reaction mixture and variations in absorbance were recorded five times at 2 min interval and finally change in absorbance per min was found (Ellman et al., 1961). Then following formula was applied to find level of AChE;

$$R=5.74\times 10^{-4}\times A/CO$$

*R* is the rate (moles) of substrate hydrolyzed/min/g of brain tissue, *A* is change in absorbance per min, and *CO* is original concentration (20 mg/ml) of tissue.

#### Assessment of Malondialdehyde (MDA) Level in Brain

The level of malondialdehyde (MDA) was determined by mixing 100 μL of brain homogenates with 1.5 ml of TBA (0.8% w/v), 1.5 ml of acetic acid (20% v/v) and 200 μL of sodium dodecyl sulfate (8% w/v). The reaction mixture was heated at 95°C for 1 h and then 5 mL of *n*-butanol was added to mixture after cooling it at room temperature. Mixture was centrifuged at 976 ×*g* for 10 min and organic layer formed at top was collected for which the absorbance was measured at 532 nm (Xian et al., 2011). The following formula was used to find the concentration of MDA in brain.

MDA (μM)=A(sample)×DF/I×ε

*I* = light path = 1 cm, 𝜀 = molar absorptivity *=* 1.56 × 10^5^ M^-10^cm^-1^ and DF = dilution factor = 21.

#### Measurement of Superoxide Dismutase (SOD) Level

The level of SOD was found by diluting brain homogenate (0.5 ml) with distilled water (1 ml) which was then added chilled ethanol (2.5 ml) and chloroform (1.5 ml). Mixture was well shaken and centrifuged for 1 min at 4°C. The supernatant was mixed with 1.2 ml of (0.025 M, pH 8.4) sodium pyrophosphate buffer, 0.3 ml of 30 μM NBT, 0.1 ml of 186 μM PMS, 0.2 ml of 780 μM of reduced nicotinamide adenine dinucleotide (NADH) and 3 ml of distilled water. The reaction mixture was incubated for 90 s at 30°C and the reaction which was initiated by addition of NADH was stopped by subsequent addition of glacial acetic acid (1 ml). Reaction mixture was vigorously stirred and then mixed with *n*-butanol by gentle shaking. Butanol layer was separated out and absorbance was measured at 560 nm against butanol blank. Amount of SOD was expressed as unit/mg of protein (Kakkar et al., 1984).

#### Measurement of Catalase (CAT) Activity

Catalase (CAT) activity was found by mixing tissue homogenate (0.1 ml) with 1.0 ml of 0.01 M phosphate buffer (pH 7.0) and 0.4 ml of 2 M H_2_O_2_. Then 2 ml of dichromate acetic acid reagent composed of 5% potassium dichromate and glacial acetic acid in ratio 1:3 was added in reaction mixture to stop the reaction and absorbance was taken at 620 nm. CAT activity was expressed as μM of H_2_O_2_ decomposed/min/mg of protein (Sinha, 1972).

#### Determination of Glutathione (GSH) Activity

Brain homogenate (0.4 ml) was mixed with 0.4 ml of 20% TCA and mixture was centrifuged at 10,000 ×*g* for 20 min at 4°C. The supernatant (0.25 ml) was then added 0.6 M DTNB (2 ml) and phosphate buffer (0.2 M, pH 8.0) was used to make final volume of 3 ml. Then absorbance was read at 412 nm against blank. Standard calibration curve was made by using different concentrations of glutathione (GSH) (10–50 μM) by dissolving in TCA (0.4 ml) and concentration of GSH in brain was expressed as μM/mg of tissue protein (Moron et al., 1979).

### Statistical Analysis

The data were expressed as mean ± SEM. Student’s *t*-test analysis was applied on data with paired comparisons and multiple comparisons were made by ANOVA followed by Dunnett’s test by using GraphPad Prism software version 7. Value of *P* < 0.05 was marked as significant.

## Results

### Percentage Yield of Plant Extract

Simple methanolic extraction of *L. stoechas* gave 220 g semi solid extract per 1,000 g of dried powdered plant material and hence % age yield was 22% w/w.

### Phytochemical Constituents

Qualitative phytochemical analysis of methanolic extract of *L. stoechas* (meL.s) showed the presence of following constituents as expressed in Table 2.

**Table 2:** Phytochemical analysis of methanolic extract of *Lavandula stoechas* (meL.s).

No.	Phytochemical constituents	Tests	Presence
1	Proteins	Ninhydrin test	++
2	Carbohydrates	Molish test	+
3	Alkaloids	Hagers’s test	++
		Wagner’s test	++
		Dragendroff’s test	+
		Mayer’s test	++
4	Glycosides	Killer-Kiliani test	+
5	Flavonoids	Alkaline reagent test	+
6	Steroids	Ring test	+
7	Terpenoids		+
8	Saponins	Foam test	+
9	Tannins	Ferric chloride test	+
10	Phenols	FC method	++
11	Quinones		-
12	Phytosterol	Liebermann–Burchard test	++
13	Terpenes	Salkowski test	+
14	Fixed oils	Spot test	-

*+, present; ++, highly present; and -, absent*.

### Quantitative Analysis of Bioactive Constituents of Methanolic Extract of *L. stoechas* (meL.s)

Total phenolic, flavonoid, and tannin contents in methanolic extract of *L. stoechas* (meL.s) were found to be 285.91 ± 0.75 mg of GAE/g of extract, 134.06 ± 0.63 mg of RE/g of extract and 149.60 ± 0.93 mg of TAE/g of dried plant extract, respectively.

### Free Radical Scavenging Activity

**FIGURE 1:**
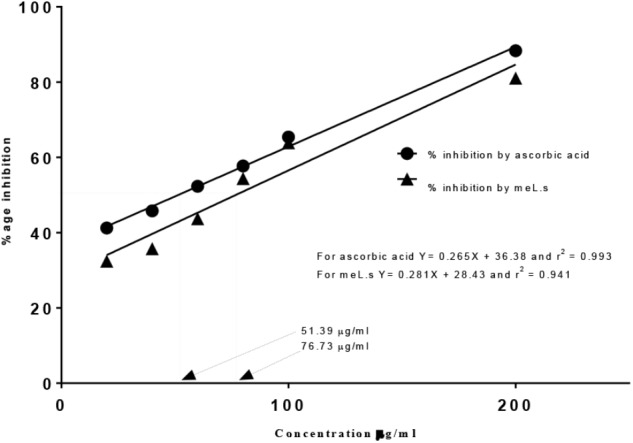
Free radical scavenging activity of meL.s and standard (ascorbic acid) along with their IC_50_ values.

Free radical scavenging activity found by DPPH assay indicated that IC_50_ value for methanolic extract of *L. stoechas* (meL.s) was 76.73 μg/ml and that of standard ascorbic acid it was 51.39 μg/ml as shown in Figure 1. Moreover, considering yield of extract, it was calculated that 1 mg of crude plant powder was equal to 220 μg of meL.s.

### Effect of Methanolic Extract of *L. stoechas* (meL.s) on Transfer Latency (TL) in EPM Paradigm

**FIGURE 2:**
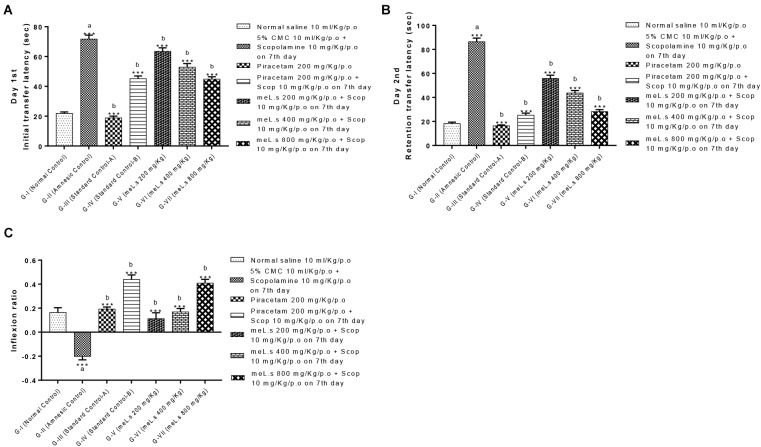
Effect of meL.s on **(A)** initial transfer latency, **(B)** retention transfer latency, and **(C)** Inflexion ratio in elevated plus maze paradigm. All values were expressed as mean ± SEM with *n* = 6 per group. One-way ANOVA followed by Dunnett’s test was applied on data set and G-II was compared with G-I (presented by sign “a” on bar) while other groups (G-III to G-VII) were compared with G-II (presented by sign “b” on bars). Value of *P* ≥ 0.05 was shown by marking “ns” while *P*-values ≤0.05, ≤0.01, and ≤0.001 were expressed as ^∗^, ^∗∗^, and ^∗∗∗^, respectively.

Initial transfer latency (ITL) was recorded on day 7th of treatment (45 min after administration of scopolamine) which reflected the learning behavior of animal while, retention transfer latency (RTL) was noted after 24 h of administration of last dose which exhibited retention of learning tasks. Inflexion ratio was calculated from transfer latencies which indicated the improvement of memories in mice. It was observed that ITL and RTL values of Group-II (amnesic control) were significantly (*P* < 0.001) high from values of Group-I (normal control) which clearly indicated the loss of memory in amnesic group. Standard control, meL.s 400 mg/kg/p.o. and meL.s 800 mg/kg/p.o. significantly (*P* < 0.001) reduced the time spent by animal in open arms as compared to Group-II which indicated improvement of memory as shown in Figure 2A. Similarly, RTL values of Groups-III to VII were significantly less (*P* < 0.001) than values of Group-II which indicated the retention of memory by animals as shown in Figure 2B. Inflexion ratios of Group-IV and VII were 0.44 ± 0.04 and 0.40 ± 0.03, respectively, which were significantly (*p* < 0.001) higher than amnesic group having IR = -0.20 ± 0.03. While Group V and VI have IR = 0.11 ± 0.05 and 0.17 ± 0.03, respectively, as shown in Figure 2C.

### Effect of Methanolic Extract of *L. stoechas* (meL.s) on Time Spent in Light and Dark Compartment

**FIGURE 3:**
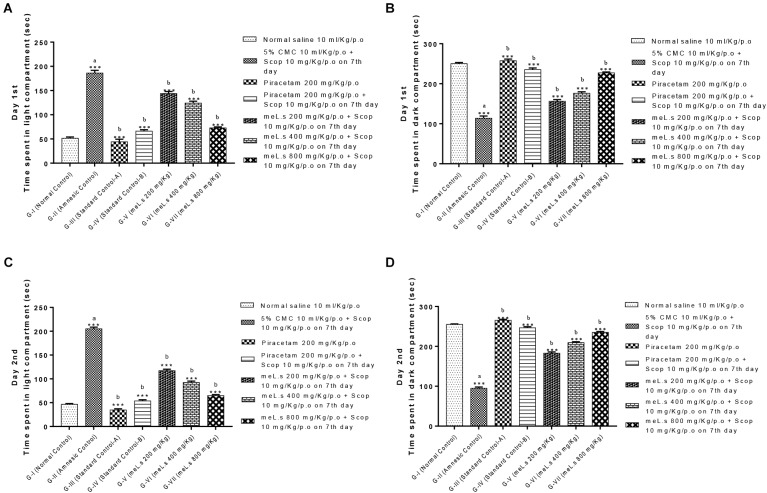
Effect of meL.s on **(A)** time spent (s) in light compartment on day 1st, **(B)** time spent (s) in dark compartment on day 1st, **(C)** time spent (s) in light compartment on day 2nd, and **(D)** time spent (s) in dark compartment on day 2nd. All values were expressed as mean ± SEM with *n* = 6 per group. One-way ANOVA followed by Dunnett’s test was applied on data set and G-II was compared with G-I (presented by sign “a” on bar) while other groups (G-III to G-VII) were compared with G-II (presented by sign “b” on bars). Value of *P* ≥ 0.05 was shown by marking “ns” while *P*-values ≤0.05, ≤0.01, and ≤0.001 were expressed as ^∗^, ^∗∗^, and ^∗∗∗^, respectively.

Effect of meL.s on time spent in both compartments of light and dark paradigm is shown in Figure 3. Animals in Group-I spent less time in light compartment and remained most of time in dark compartment. Time spent by animals in light compartment was significantly increased (*P* < 0.001) in Group-II as compared to Group-I animals. Similarly, animals in Groups III-VII spent most of the time in dark arena both on first and second day which indicated that they significantly (*P* < 0.001) improved memory as compared to amnesic group.

### Effect of Methanolic Extract of *L. stoechas* (meL.s) on Number of Hole Pokings in Hole Board Paradigm

**FIGURE 4:**
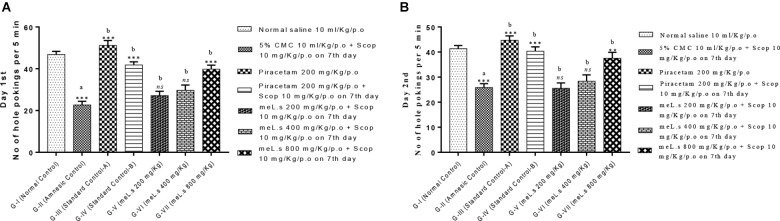
Effect of meL.s on **(A)** no of hole pokings by mice on day 1st and **(B)** no of hole pokings by mice on day 2nd. All values were expressed as mean ± SEM with *n* = 6 per group. One-way ANOVA followed by Dunnett’s test was applied on data set and G-II was compared with G-I (presented by sign “a” on bar) while other groups (G-III to G-VII) were compared with G-II (presented by sign “b” on bars). Value of *P* ≥ 0.05 was shown by marking “ns” while *P*-values ≤0.05, ≤0.01, and ≤0.001 were expressed as ^∗^, ^∗∗^, and ^∗∗∗^, respectively.

It has been observed that no of hole pokings by mice in amnesic group was significantly (*P* < 0.001) reduced as compared to normal control group which indicated induction of amnesia. However, Groups-IV and VII significantly (*P* < 0.001) increased no of hole pokings as compared to Group-II. Groups V–VI produced non-significant changes in hole pokings both at day 1st and 2nd. All details are shown in Figure 4 which indicated that meL.s 800 mg/kg/p.o. was more effective than both of its lower doses.

### Effect of Methanolic Extract of *L. stoechas* (meL.s) on Concentration of Acetylcholinesterase (AChE) in Mice Brain

**FIGURE 5:**
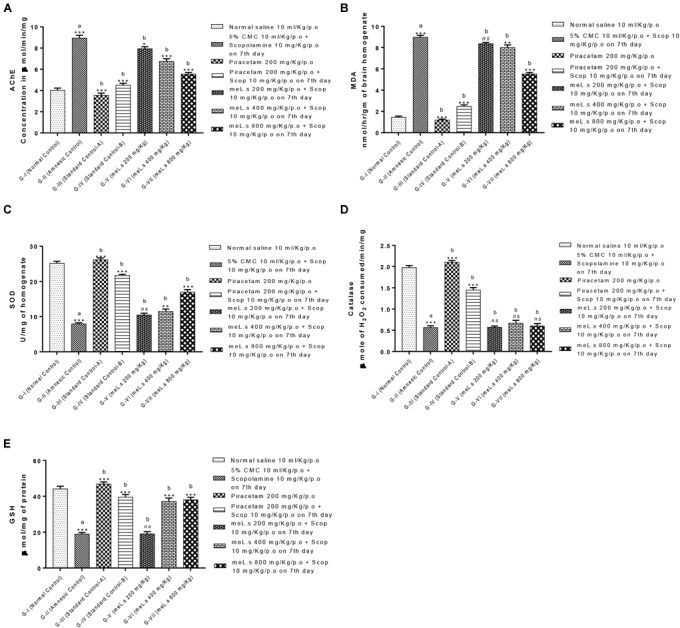
Effect of meL.s on concentration of **(A)** acetylcholinesterase (AChE), **(B)** MDA, **(C)** SOD, **(D)** CAT, and **(E)** GSH in brain homogenate. All values were expressed as mean ± SEM with *n* = 6 per group. One-way ANOVA followed by Dunnett’s test was applied on data set and G-II was compared with G-I (presented by sign “a” on bar) while other groups (G-III to G-VII) were compared with G-II (presented by sign “b” on bars). Value of *P* ≥ 0.05 was shown by marking “ns” while *P*-values ≤0.05, ≤0.01, and ≤0.001 were expressed as ^∗^, ^∗∗^, and ^∗∗∗^, respectively.

Group-II animals significantly (*P* < 0.001) increased the level of AChE as compared to normal control animals. However, pretreatment of animals with standard drug piracetam and plant extract in different doses showed marked reduction in level of AChE. Among treatment groups meL.s 800 mg/kg/p.o. caused maximum reduction in the level of AChE which was significantly (*P* < 0.001) less than the amnesic group. The details are given in Figure 5A.

### Effect of Methanolic Extract of *L. stoechas* (meL.s) on MDA, SOD, CAT, and GSH Levels in Mice Brain

It was observed that administration of scopolamine to Group-II animals significantly (*P* < 0.001) increased the level of MDA in brain while SOD, CAT, and GSH levels were declined in comparison to normal control group. A significant (*P* < 0.001) reduction in MDA and elevation in SOD, CAT, and GSH level was observed in animals treated with standard drug piracetam in comparison to Group-II animals. Pretreatment of animals with meL.s 800 mg/kg/p.o. significantly decreased the MDA contents of mice brain while SOD and GSH were significantly (*P* < 0.001) improved as compared to groups treated with same extract in low doses but CAT level was non-significantly improved by it. From Figure 5 it is clear that meL.s 200 mg/kg/p.o. did not increase the level of SOD, GSH, and CAT while meL.s 400 mg/kg/p.o. produced less significant (*P* < 0.05) results as compared to amnesic group. Similarly, 800 mg/kg/p.o. non-significantly improved the CAT level. The detailed results of biochemical markers are shown in Figures 5B–E.

## Discussion

Neurodegeneration in the brain, initially results into loss of short term memory (Burns and liffe, 2009) which progresses toward disorientation speech, mood swing, social withdrawals, altered behavioral patterns and ultimately death (Todd et al., 2013) due to loss of cognition over a time span of a decade or more (Alzheimer’s Association Report, 2017). Cholinergic hypothesis best narrates the pathogenesis of dementia by loss of cholinergic innervations in frontal cortex, cingulated gyrus and hippocampus of the brain (Wenk, 2003). Similarly, accumulation of β-amyloid protein and extra neuronal plaque formation due to severe oxidative damage (Da Silva Filho et al., 2017; Omar et al., 2017) are main causes of memory loss (Hardy and Higgins, 1992; Wilson and Binder, 1997; Da Silva Filho et al., 2017). It has been roughly estimated that oxidative stress is responsible for pathogenesis of more than hundred diseases (McCord, 2000) including dementia (Pratico, 2008). Anticholinergic drugs especially scopolamine disrupt both short term and working memory by muscarinic blockade of neurons and hence can be employed for induction of amnesia in rodents to evaluate antiamnesic activity of an agent (Baxter et al., 2013). In contrary, agents enhancing cholinergic neuronal activity in brain and those preventing oxidative stress in brain can be used to prevent progression of dementia (Rabiei et al., 2014; Rajesh et al., 2017). Currently, very few drugs are available for the management of dementia including piracetam, galantamine, donepezil, memantine and rivastigmine which only provide symptomatic relief and are associated of severe toxicity. They do not prevent the progression of underlying pathophysiological aspects of dementia (Salomone et al., 2012). Bioactive herbal constituents (Howes et al., 2003) demonstrating anti-amnesic properties are of great interest of researchers in current era to explore successful remedy of dementia (Houghton and Howes, 2005). A toxicity free profile and sustained long lasting neuroprotective benefits of herbal remedies are empirical evidence for the best therapeutic applications of natural plants in the management of memory related disorders (Omar et al., 2017). Considering potential beneficial effects of *Lavandula stoechas* (L) in management of dementia in traditional medicinal practice (Nadkarni, 1996), this study was planned to explore its active constituents responsible for anti amnesic activity. The current research paper is initial finding of this series. In this investigation EPM, light and dark test and hole board paradigms were used for behavioral observations to reach the conclusion as proposed by Hossain and Uma Devi (2001).

Behavioral studies using EPM paradigm indicated that methanolic extract of *L. stoechas* showed dose dependent decrease in transfer latencies (decrease latency means improvement of learning tasks) and increased the inflexion ratio (a hallmark of improvement in retaining learned tasks) in comparison to amnesic group. Currently, EMP is widely employed paradigm in assessment of memory and learning tasks in rodents (Barez-Lopez et al., 2017) and considered reliable method of assessment of memory (Chauhan and Chaudhary, 2012). Pre treatment of animals with plant extract prior to administration of scopolamine prevented the impairment of learning and retaining capabilities which indicates the effectiveness of *L. stoechas* in memory build up. Similarly, findings of light dark paradigm illustrate that extract treated animals retained their learned ability of spending most of time in dark area while G-II animals (scopolamine treated) lost their memory to go into dark compartment and hence lived most of the time in light area. This supported the effectiveness of memory enhancing effect of plant along with efficiency of this paradigm in evaluation of memory tasks (Barry et al., 1987). Hole board paradigm used for behavioral analysis was based upon concept that increased no of hole pokings by mice retained their exploration behavior while scopolamine impaired their memory of exploration (Durcan and Lister, 1988). Thus it was observed that standard drug piracetam and plant extract in high doses increased the no of hole pokings by mice as compared to scopolamine treated mice. Investigation of memory enhancing effect of *L. stoechas* through behavioral analysis was further supported by evaluation of biochemical markers in brain homogenates of mice.

Acetylcholine is degraded by AChE at the level of synaptic cleft which diminishes cholinergic transmission (Ballard et al., 2005). An agent enhancing the level of AChE will impair memory by reducing acetylcholine levels as scopolamine did in amnesic group. In contrary, standard drug piracetam and methanolic plant extract (800 mg/kg/p.o.) retained the memory of mice as observed by behavioral studies by significantly (*P* < 0.001) lowering the level of AChE in brain (Figure 5A). Presence of alkaloids and flavonoids (134.06 ± 0.63 mg/g) in *L. stoechas* supported the acetyl cholinesterase activity of plant extract (Ma and Gang, 2008). Moreover, anti-oxidant studies suggested that plant extract also prevented the brain from oxidative stress (Pratico, 2008) by raising the level of SOD, GSH and CAT as shown in Figures 5B–E. Brain is highly susceptible to be damaged by oxidizing agents because high oxygen consumption, low GSH levels and polyunsaturated fat deposition in brain damage the neurons in brain (Mamelak, 2007; Sonnen et al., 2008). Exposure of brain with hydrogen peroxide results into production of several enzymes like β-secretase and γ-secretase which cleave amyloid precursor protein into amyloid β-peptide. Accumulation of amyloid β-peptide in brain is hallmark of loss of memory (Butterfield, 2002; Tong et al., 2005). Similarly, lipid peroxidation in brain elevates the level of MDA in brain which suggested loss of memory due to oxidative stress (Sultana et al., 2013). Thus, anti-oxidants protect the brain from this damage by scavenging free radicals (Omar et al., 2017). *In vitro* antioxidant activity of plant extract as observed by DPPH method ensured that it exhibited free radical scavenging activity (Figure 1). Total phenols were estimated to be 85.91 mg/g plant extract which were supposed to scavenge free oxygen, hydrogen peroxide, superoxide, and hydroxyl radicals (Pereira et al., 2009) and prevented the brain from oxidative stress. Scopolamine damaged the memory by depleting natural antioxidants present in brain, i.e., SOD, GSH, and CAT (El-Sherbiny et al., 2003) but current findings clearly declared *L. stoechas* a strong anti oxidant which reduced the level of MDA and increased SOD, GSH, and CAT levels in mice brain. Elevation of SOD and CAT prevented the damage caused by superoxide radicals and H_2_O_2_ respectively (Bhattacharjee et al., 2015) while GSH scavenged free radicals in brain proteins (Farombi et al., 2000). Based upon concluding results of current study, it is increasingly evidenced that antioxidant supplementation improves cognition on one hand and slows down the progression of dementia on other side.

## Conclusion

It is concluded that dementia is linked with oxidative stress and loss of cholinergic innervations in brain neurons. *L. stoechas* could prove helpful in attenuation of dementia as it reduces oxidative burden of neurons and decreases neuro-degradation of cholinergic transmission in mice brain. This research is first finding of the series and further studies are in progress in our lab to reach a bio molecule of *L. stoechas* actually responsible for anti amnesic activity along with an appropriate mechanism of action.

## Author Contributions

AM conducted the experimental work. MA and RA proposed the study design, supervised the experimental work, and guided in writing manuscript.

## Conflict of Interest Statement

The authors declare that the research was conducted in the absence of any commercial or financial relationships that could be construed as a potential conflict of interest.
